# Animal Models of Alcohol’s Motivational Effects

**Published:** 2000

**Authors:** Christopher L. Cunningham, Tara L. Fidler, Katherine G. Hill

**Affiliations:** Christopher L. Cunningham, Ph.D., Tara L. Fidler, Ph.D., and Katherine G. Hill, Ph.D., are affiliated with the Department of Behavioral Neuroscience and Portland Alcohol Research Center, Oregon Health Sciences University, Portland, Oregon

**Keywords:** animal model, AOD (alcohol or other drug)-seeking behavior, motivation, self administration of drugs, operant conditioning, learning, memory, place conditioning, taste conditioning

## Abstract

Alcohol’s positive and negative motivational effects are believed to be important influences on alcohol-seeking behavior and, therefore, key factors among the many and varied causes of alcohol abuse and dependence. Alcohol’s positive effects, such as enhanced mood, and negative effects, such as hangover, are considered important factors in motivating drinkers to increase or decrease their drinking. Scientists have developed a variety of animal behavioral models to study alcohol’s motivational effects. These models include “self-administration models,” in which the animal controls the exposure to alcohol, and “conditioning models,” in which the researcher controls the animal’s exposure to alcohol. Such models have been used to study the influence of genetic differences on sensitivity to alcohol’s positive and negative motivational effects, the brain mechanisms underlying alcohol’s motivational effects, as well as relapse and craving.

The causes of excessive alcohol use and alcoholism are complex, reflecting the interaction of a wide range of genetic, environmental, sociocultural, and experiential factors. Among these factors, alcohol’s positive and negative motivational effects often stand out in theoretical analyses of alcohol-seeking behavior. Researchers believe, for example, that alcohol’s positive effects on mood may motivate a person to drink more, and that likewise, alcohol’s negative effects, such as hangover, may motivate a person to drink less. These effects are considered important factors in determining whether people who drink will continue to consume alcohol and increase their intake of alcohol over time (Tabakoff and Hoffman 1988).

Positive motivational effects produced by alcohol can include increases in pleasurable states (e.g., elation and euphoria) as well as the alleviation of unpleasant states such as those produced by stress, anxiety, or physical dependence and withdrawal. Negative motivational effects produced by alcohol may include increases in unpleasant states (e.g., dysphoria, illness, hangover) or reductions in pleasurable states (e.g., reduced elation). Presumably, individual differences in sensitivity to such motivational effects can either facilitate or inhibit the development of excessive drinking patterns characteristic of alcohol abuse and alcoholism.

Given the theoretical significance placed on alcohol’s motivational effects, scientists have developed a variety of animal behavioral models to assess those effects. Although many different animal species have been examined, most studies have used monkeys or rodents (e.g., rats and mice). Many of the initial efforts in this area were heavily criticized for failing to meet the formal criteria proposed for “animal models of alcoholism” (e.g., Lester and Freed 1973; Cicero 1979). For example, few animal models have shown sustained voluntary intake of alcohol at levels that produce a withdrawal syndrome when the alcohol is removed. Most investigators in the field, however, no longer view animal models as attempts to create “alcoholism.” Rather, these models are now used primarily to characterize alcohol’s motivational effects, with the hope that this knowledge will shed light on the roles these motivational effects play in developing and maintaining excessive drinking in humans. Researchers also use these models to study neurobiological and genetic mechanisms underlying alcohol’s motivational effects and to develop pharmacological and behavioral interventions to alter those effects.

The purpose of this article is to offer a brief overview of the animal behavioral models currently used to study alcohol’s motivational effects. This overview will focus on models that directly measure seeking or avoidance of alcohol or alcohol-paired stimuli (e.g., a flavored solution that is provided with alcohol). Due to limited space we cannot discuss certain well-studied models in which alcohol’s motivational effects are inferred from its ability to alter the effects of other motivational variables such as rewarding brain stimulation (Kornetsky et al. 1988), stress (Pohorecky 1981), or anxiety (Koob and Britton 1996).

The models described here are separated into two major categories based on whether exposure to alcohol is usually controlled by the animal or by the experimenter. Models in the first category are described under the heading “self-administration models” whereas those in the second category are discussed under the heading “conditioning models.” The discussions will focus on the general rationale behind each model, key research findings, and issues related to the interpretation of the models.

## Self-Administration Models

In self-administration models, animals control their alcohol intake and thus determine the amount (dose) and temporal pattern of their intake. In conditioning models, the experimenter administers a fixed dose of the drug, independent of the animal’s behavior. We will discuss two types of self-administration models, home cage drinking and operant conditioning. They can generally be distinguished from each other on the basis of the behavior required to obtain alcohol (e.g., approaching a drinking bottle in the home cage vs. pressing a bar in a testing chamber) and the route of alcohol administration (oral vs. infusion via surgically implanted tubes). We will first consider those models involving measures of intake or preference in the home cage and then describe experimental procedures that involve operant conditioning techniques.

### Home Cage Drinking and Preference

One of the oldest approaches to studying avidity or preference for water-soluble drugs like alcohol is simply to measure the volume consumed when a drinking bottle containing the drug solution is placed in the home cage (e.g., Richter and Campbell 1940). Although alcohol is sometimes the only fluid available, it is more common to offer a choice between alcohol and water or among several alternative solutions (e.g., several different concentrations of alcohol). When animals are given a choice of solutions, the proportion of alcohol intake relative to total intake (i.e., preference ratio) is frequently used to characterize the animal’s behavior. In many experiments, fluid bottles are available 24 hours per day (i.e., continuous-access procedures). In some cases, however, alcohol may be available only for short periods of time each day (i.e., limited-access procedures). Whether researchers use continuous or limited access procedures usually depends on concerns over the pattern of intake over time. With long access periods (e.g., 24 hours) subjects may distribute their alcohol consumption in small, widely spaced bouts that do not necessarily produce appreciable or sustained brain alcohol levels. In contrast, limited-access procedures can encourage relatively high alcohol intake in a short period of time (Marcucella 1989).

Animals, like most humans, will not ingest large volumes of a highly concentrated alcohol solution the first time it becomes available to them. Rodents in particular are well known to be cautious about consuming novel-tasting substances (a phenomenon called neophobia). Thus, investigators have developed a variety of “tricks” for initiating alcohol intake with the goal of establishing intake levels that allow the animal to experience alcohol’s motivational effects. One common strategy is to introduce alcohol at a relatively low concentration and to gradually increase the concentration over time. Another strategy is to mix the alcohol with a highly preferred flavor, such as sucrose or saccharin, whose concentration may be gradually reduced over time. Food and fluid deprivation have also been used to encourage alcohol intake, although these manipulations raise important interpretative concerns that we will discuss later.

Home cage drinking experiments have been useful for characterizing genetic differences in alcohol intake and preference across different strains of rats and mice (e.g., Li and Lumeng 1984; McClearn and Rodgers 1959). Moreover, observations of home cage alcohol intake have been used successfully in the selective breeding of both rats and mice for high and low alcohol intake and preference (e.g., Lumeng et al. 1995), providing further evidence for a genetic influence on this behavior. Currently, several research groups are using the home cage drinking model to map and identify specific genes that control alcohol intake (e.g., Phillips et al. 1998).

Researchers have also used home cage drinking procedures to study the impact of various pharmacological pre-treatments on alcohol intake and preference. For example, recent clinical trials found that alcoholism treatment outcomes can be improved with administration of a drug (i.e., naltrexone) that interferes with brain receptors which normally react to opiate drugs like heroin and morphine (e.g., O’Malley et al. 1992; Volpicelli et al. 1992). These clinical trials were inspired, in part, by findings from home cage drinking studies showing that pretreatment with various opiate antagonist drugs suppressed alcohol intake in animals (e.g., Reid and Hunter 1984). Further research may increase our understanding of the brain systems mediating alcohol intake and identify potential pharmacological therapies for reducing alcohol intake.

### Operant Conditioning

Theoretical analyses of alcohol self-administration have distinguished between “appetitive” and “consummatory” processes involved in the regulation of alcohol intake (e.g., Samson and Hodge 1996). Appetitive processes control alcohol-seeking behavior, that is, they motivate and direct behavior toward sources of alcohol and they influence the initiation of alcohol consumption. Once drinking has begun, however, appetitive processes interact with consummatory processes, which are more directly related to maintenance and termination of drinking. Although both processes presumably affect home cage alcohol drinking, home cage experiments typically focus on consummatory processes, as indexed by response measures like total volume consumed. In contrast, operant conditioning studies, which use separate testing cages where access to alcohol is contingent upon the animal’s behavior (e.g., pressing a bar), allow greater emphasis on the role played by appetitive processes because one can separate alcohol-seeking behavior from alcohol consumption.

In operant self-administration experiments, which are based on procedures originally developed by B. F. Skinner (1938) using food reward, access to alcohol is contingent upon completion of a specific response (e.g., pressing a bar) or sequence of responses (e.g., pressing a bar four times in a row). Thus, one can measure alcohol-seeking (e.g., bar press latency or rate) in addition to the amount of alcohol consumed. The ability to measure both may be especially useful in situations where drug ingestion produces sensory or motor effects that directly interfere with continued ingestive behavior.

In operant procedures, the experimenter can vary how hard the animal must work to obtain alcohol, how frequently alcohol will be available, and how much alcohol can be consumed each time the response requirement is completed (i.e., the “schedule” of alcohol reinforcement). All of these variables have been shown to influence the rate of operant response as well as the intake of alcohol (see review by Meisch 1977). For example, when the operant response requirement is minimal (e.g., one bar press earns brief access to alcohol), daily alcohol intakes in an operant procedure are similar to those seen in a home cage drinking procedure. However, even a relatively minor increase in the response requirement (e.g., from one to four consecutive bar presses) will produce a reduction in total alcohol intake (Samson and Hodge 1996).

In many operant studies, rats are given daily limited-access (e.g., 30-minute) sessions in which completion of the bar press response requirement is repeatedly interspersed with brief periods of alcohol access. Animals in this situation typically exhibit a high response (i.e., bar press) rate at first, although that rate decreases slightly over time before terminating abruptly after 10 to 15 minutes (e.g., Samson 1986). One of the difficulties in interpreting behavior under these conditions is that response rates and intakes measured during later parts of the session may be influenced by the cumulative effects of the alcohol ingested. Recently, Samson and colleagues (1998, 1999*b*) have addressed this problem by using a procedure that more completely separates alcohol seeking from alcohol consumption. In this procedure, completion of the bar press requirement is followed by only one relatively long (20 minutes) fluid access period in each session. When the number of bar presses required to gain access to the drinking tube was doubled across consecutive sessions, rats receiving 10 percent alcohol showed increases in bar pressing similar to rats receiving 3 percent sucrose, even though sucrose intakes were consistently higher than alcohol intakes. This finding implies a dissociation between operant response measures and intake measures of alcohol’s motivational effects, suggesting that this experiment will prove useful in identifying variables that selectively influence appetitive or consummatory processes.

Although the alcohol is consumed orally in most self-administration studies, several studies show that animals will perform desired behaviors when the reward is an injection of alcohol directly into the stomach, blood, or brain via surgically implanted tubes (e.g., Deneau et al. 1969; Gatto et al. 1994; Smith et al. 1976). The principal advantage of these techniques is that they allow investigators to assess the post-absorptive motivational effects of alcohol in the absence of orosensory effects (e.g., taste, burning sensation in mouth or throat) that might complicate the interpretation of results. Studies in which the animal’s behavior causes a small amount of alcohol to be injected directly into the brain have the additional advantage of allowing researchers to localize specific brain areas that mediate alcohol reward. The value of examining nonoral routes of administration is nicely illustrated by a recent study in which alcohol was injected directly into the bloodstream whenever mice poked their nose in a hole in the chamber wall (Grahame and Cunningham 1997). This study compared self-injection of alcohol in two mouse strains (C57BL/6 and DBA/2) that are well known to differ in alcohol intake in home cage drinking and oral operant conditioning procedures. In contrast to the usual finding of better performance in C57BL/6 mice, both strains performed similarly when nose poking produced intravenous infusions of alcohol, suggesting that oral self-administration by DBA/2 mice is normally suppressed by aversive orosensory effects of alcohol.

### Interpreting Self-Administration Models

Oral self-administration models seem valid as models for humans because human alcohol users typically drink alcohol under circumstances in which they control the amount consumed and the pattern of consumption. However, self-administration procedures pose unique interpretive challenges for scientists trying to understand the nature and source of the motivational effects that influence self-administration. Self-administration theories often emphasize the hypothesized role of alcohol’s pharmacological effects, such as an increase in pleasant (or unpleasant) feelings or a decrease in stress, anxiety, or the effects of withdrawal. However, research shows that oral self-administration is influenced by many nonpharmacological variables, including taste, palatability, and the caloric value of the alcohol. Thus, it is possible that individual differences or changes in oral self-administration may be related more to variations in sensitivity to alcohol’s orosensory effects or its caloric value than to its pharmacological effects. Moreover, manipulations designed to increase the animals’ exposure to alcohol’s pharmacological effects can complicate interpretation when those manipulations also affect taste (e.g., adding sweetener) or caloric need (e.g., food deprivation).

Researchers can address these issues by carefully monitoring the amount of alcohol consumed and the pattern of consumption. Information on the number, size, and temporal distribution of drinks during a day is more useful than the total volume of alcohol consumed per day in determining whether the pharmacological effect is a plausible source of motivation for self-administration. Arguments in favor of the interpretation that the motivation to drink alcohol occurs because of its pharmacological effect are also much stronger when they can be supported by data showing blood or brain alcohol levels in a range known to have behavioral or physiological effects. However, observing large drinking bouts or high levels of alcohol does not eliminate the influence of orosensory or caloric factors. As noted earlier, self-administration of alcohol via nonoral routes (e.g., intravenous) offers one approach to assessing alcohol’s motivational properties in the absence of its orosensory effects. In addition, the influence of alcohol’s caloric value is presumably reduced by procedures that do not involve food deprivation (Samson 1986).

Sweeteners, such as sucrose or saccharin, are often added to alcohol solutions to facilitate initiation of alcohol self-administration (e.g., Samson 1986). Not surprisingly, sweetened alcohol is consumed in greater volumes than unsweetened alcohol (e.g., Samson et al. 1999*a*). This can be explained in several ways. For example, the taste of the sweetener may mask alcohol’s aversive taste or produce positive motivational effects that offset alcohol’s aversive orosensory effects. With sucrose, postingestion caloric effects provide an additional source of motivation. It has also been suggested that sucrose may alter alcohol absorption (Roberts et al. 1999, but see Gauvin 1999; Czachowski et al. 1999). Of course, by increasing the overall intake of alcohol, a sweetener will result in a greater pharmacological effect on the animal. In most self-administration studies involving sweetened alcohol, it is difficult to separate these possibilities. However, recent studies by Heyman and colleagues (e.g., Heyman et al. 1999) indicate that self-administration of sweetened alcohol is controlled, at least in part, by alcohol’s pharmacological effects. For example, rats given a simultaneous choice between responding to sweetened alcohol or an isocaloric non-drug nutrient, which provides the same number of calories as alcohol (e.g., Polycose), worked harder to maintain alcohol intake than to maintain intake of the isocaloric nutrient when bar press response requirements were increased. Although these studies do not eliminate influence of taste and calories, they offer strong evidence of a role for alcohol’s pharmacological effects in self-administration of sweetened alcohol.

## Conditioning Models

Learning and memory play critical roles in the appetitive processes that contribute to the regulation of alcohol consumption. Researchers also believe that learning and memory contribute to craving and the phenomenon of relapse after long periods of abstinence. Because a complete discussion of the roles played by learning and memory is beyond the scope of this paper, we discuss one particular type of learning that provides the basis for two conditioning models of alcohol’s motivational effects. Specifically, we describe two models derived from the methods and conceptual framework originally developed by Ivan Pavlov (1927/1960). These models are based on the premise that individuals can learn associations between drugs and stimuli that predict drug administration. In the language of Pavlovian conditioning, drug-predictive stimuli are called “conditioned stimuli,” or CSs, whereas drug effects are called “unconditioned stimuli,” or USs. Potential CSs for alcohol include its taste and odor as well as external cues (e.g., visual, auditory) related to the setting in which it is consumed. The USs for alcohol encompass the range of its pharmacological effects (e.g., thermal, cardiovascular, sedative), including its motivational effects (e.g., euphoria, dysphoria, antianxiety). As a result of CS-drug associations, CSs acquire the ability to elicit new responses, to alter the original response to the drug, and to change the individual’s motivational state in the absence of the drug (Cunningham 1993, 1998).

The two models under consideration, place and taste conditioning, differ primarily in the nature of the CS paired with alcohol and the type of response used to index learning. In place conditioning, distinctive environmental cues (e.g., visual, tactile) are paired with drug effects and the experimenter later measures the animal’s approach to or withdrawal from those cues. In taste conditioning, novel taste cues are paired with drug exposure and the experimenter measures subsequent intake or preference for the flavored food or fluid.

### Place Conditioning

In a typical place conditioning experiment, rats or mice are trained in a specially designed apparatus that permits presentation of different visual, tactile, auditory, or olfactory stimuli in spatially distinct locations. For example, the apparatus might consist of two attached compartments that differ in the texture of the floors (smooth vs. rough) and the brightness of the walls (black vs. white). Animals are usually given a series of trials over several days in which one set of stimuli is consistently paired with alcohol exposure and the second set of stimuli is not. On a subsequent test day, animals are placed in the apparatus (usually in a drug-free state) and given free access to both sets of distinctive stimuli. The amount of time spent in the presence of each set of stimuli is recorded as a measure of the animal’s conditioned preference for or aversion to the alcohol-associated stimuli. For example, if an animal spends a relatively greater portion of time in the alcohol-paired context, researchers believe that this behavior reflects alcohol’s positive motivational effects. Spending more time in the opposite (nondrug) context is usually interpreted as avoidance of the alcohol-associated stimuli and a reflection of alcohol’s negative motivational effects. Of course, researchers use various control procedures to ensure that such outcomes reflect learning about the drug’s motivational effects and not just innate preferences or aversions for the CSs (Cunningham 1993).

Researchers have used the place conditioning paradigm to study the motivational effects of a wide variety of abused drugs, including alcohol (see Tzschentke 1998 for a recent review of place conditioning literature). Alcohol’s ability to produce conditioned place preference (or aversion) depends on a number of variables, including species and strain of animal, the dose and route of administration of alcohol, and the animal’s past history of alcohol exposure. For example, rats and mice exhibit different sensitivities to alcohol’s rewarding effects (Cunningham et al. 1993). Although most studies with rats have shown alcohol-conditioned place aversion (Sherman et al. 1988), alcohol-conditioned place preference has been shown in a variety of mouse strains (e.g., Cunningham et al. 1991, 1992). The overall pattern of findings suggests that rats and mice may be differentially sensitive to the positive and negative motivational effects of alcohol. Even among mouse strains, however, there is considerable variation in alcohol place conditioning, with some strains showing strong conditioned preferences and others showing little or no effect (Cunningham 1995).

Because of the difficulty in reliably demonstrating alcohol-conditioned place preference in rats, researchers have rarely used the rat place conditioning model to study genetic differences or neurobiological mechanisms underlying alcohol’s motivational effects. In one of the few studies of genetic differences, rats selectively bred to prefer alcohol in a home cage drinking model (P rats) were found to develop weaker alcohol-conditioned place aversions than rats bred to avoid alcohol (NP rats) (Stewart et al. 1996). Although P rats fail to display alcohol-conditioned place preference, the finding of a weaker conditioned place aversion compared with NP rats is generally consistent with the fact that P rats consume more alcohol than NP rats in home cage self-administration studies.

Since the discovery of alcohol-conditioned place preference in mice, there has been an increased interest in using this model to study genetic and neurobiological influences on alcohol reward. In light of earlier studies showing the effects of opiate antagonists on alcohol self-administration (i.e., opiate antagonists reduced alcohol intake), recent studies of the effects of opiate antagonists on alcohol-conditioned place preference are especially interesting. These studies have shown that pre-treatment with naloxone (an opiate receptor antagonist) on the day of testing has a detrimental effect on maintaining alcohol-conditioned place preference in mice (Cunningham et al. 1998). This finding raises the possibility that environmental cues associated with alcohol’s effects may be able to elicit conditioned changes in endogenous opiates (e.g., endorphins) that normally maintain conditioned place preference via activity at opiate receptors. This interpretation is generally consistent with previously reported effects of opiate antagonists on alcohol self-administration (i.e., reduced alcohol intake), suggesting that opiate antagonist effects in the self-administration model reflect interference with Pavlovian components of the appetitive processes regulating alcohol intake.

### Taste Conditioning

In taste conditioning studies, drug effects (USs) are typically paired with ingestion of a novel-tasting food or liquid (CS). The drug is most often given by injection, although it is sometimes mixed together with the taste CS. The effects of taste-drug pairings are evaluated by measuring subsequent intake or preference for the CS in the absence of the drug. Presumably, drugs producing positive motivational effects should increase intake or preference for the paired CS, whereas drugs producing aversive effects should decrease CS intake or preference. Much of the early work with the taste conditioning model emphasized its utility for detecting aversive motivational effects of treatments such as exposure to X-rays or illness-inducing drugs like lithium chloride (Riley and Tuck 1985). Thus, it was somewhat surprising when researchers found that injections of commonly abused drugs like alcohol, amphetamine, and morphine also reduced intake of paired-taste CSs, raising the possibility that these drugs produce aversive motivational effects (Hunt and Amit 1987).

The literature now consistently shows the development of conditioned avoidance of taste solutions that have been paired with moderate-to-high dose alcohol injections in both rats and mice. As with the models discussed earlier, research on alcohol taste conditioning has addressed genetic influences and neurobiological mechanisms of alcohol’s aversive effect. For example, selectively bred P rats are more resistant to alcohol-induced conditioned taste aversion than NP rats (Froehlich et al. 1988). The study of alcohol-conditioned taste aversion is also contributing to the ongoing search for specific genes that influence alcohol’s motivational effects (Risinger and Cunningham 1998) and to the identification of neurotransmitters underlying those effects (e.g., Risinger et al. 1999; Sklar and Amit 1977).

Although most taste conditioning studies have demonstrated conditioned aversion in both rats and mice, a few studies suggest that taste-alcohol pairings can sometimes establish a taste preference. For example, studies have shown that pairing a distinctive flavor with either self-administered alcohol in an operant procedure (Cunningham and Niehus 1997) or a low-dose alcohol infusion directly into the stomach (Deems et al. 1986; Sherman et al. 1983) establishes a flavor preference in food-deprived rats. Researchers have also demonstrated a preference among P rats for an alcohol-paired flavor by using a procedure in which drinking a flavored solution produced a direct infusion of alcohol into the stomach (Waller et al. 1984). Moreover, non-deprived rats were found to develop a preference when the flavor was simply mixed in a low-concentration alcohol solution that was continuously available in the home cage (Mehiel and Bolles 1984). In most of these studies, the researchers’ explanations for the observed preference focused on alcohol’s caloric content (i.e., the animals preferred the alcohol solutions because these solutions, with their higher caloric content, could help compensate for the food deprivation.) However, in experiments that show preference for flavors paired with relatively high blood alcohol levels, the influence of pharmacological effect is not easily dismissed (Waller et al. 1984).

### Interpretation of Conditioning Models

At first glance, one might question the overall relevance of conditioning models, in which subjects do not “voluntarily” ingest alcohol. However, just as self-administration models are intended to capture only one component of the behavioral processes contributing to alcoholism, conditioning models focus on a subset of the learning and memory processes thought to influence alcohol seeking and self-administration. More specifically, the conditioned motivational effects captured by these models are assumed to constitute a major part of the “appetitive” processes regulating alcohol self-administration. Thus, alcohol-induced conditioned preferences for environmental locations or flavors associated with alcohol exposure would be expected to increase alcohol seeking and contact with sources of alcohol, whereas alcohol-induced conditioned aversions would be expected to encourage withdrawal from and avoidance of alcohol sources. Conditioned physiological and motivational responses have played important roles in learning-based theories of relapse and in the development of cue-exposure treatments for alcoholism, in which patients are repeatedly exposed while sober to stimuli that have previously been paired with alcohol (Drummond et al. 1990).

Conditioning models offer several methodological advantages for studying alcohol’s motivational effects. Because the experimenter specifies the CS and its temporal relationship to alcohol administration, these models are especially well suited for analyzing the roles played by various types of stimuli that signal imminent exposure to alcohol’s intoxicating effects. In addition, the experimenter has greater control over the dose, duration, and temporal pattern of alcohol exposure; can examine the effects of alcohol doses that are not normally self-administered; and can conduct tests in the complete absence of alcohol. Nonoral routes of alcohol administration are also commonly used in conditioning models, eliminating concerns about alcohol’s aversive orosensory effects. Another advantage of these models is that evidence of alcohol’s motivational effects can often be obtained after only a few exposures to alcohol, whereas self-administration studies usually require a lengthy initiation period.

From a conceptual standpoint, the ability of conditioning models to detect either preference or aversion represents another important advantage. However, our understanding of alcohol’s bivalent effects in these models remains incomplete. For example, we cannot yet explain why rats and mice differ in their apparent sensitivity to alcohol’s positive motivational effects in the place conditioning model, even though both species show a generally similar sensitivity to aversive effects in the taste conditioning model. Moreover, we have difficulty explaining how the same dose of alcohol can produce both a positive motivational effect in one model (e.g., place preference in mice) and a negative motivational effect in the other model (e.g., taste aversion in mice). Ultimately, an understanding of these “paradoxes” is critical, both for integrating data from conditioning and self-administration models and applying other findings to humans.

## Summary and Conclusions

This article has briefly described a few examples of two general types of animal models commonly used to study alcohol’s motivational effects. All of the behavioral procedures described here have proved useful in detecting differences among inbred or selectively bred rodents, consistent with the hypothesis that genetically determined individual differences affect sensitivity to alcohol’s positive and negative motivational effects (Tabakoff and Hoffman 1988). All of these procedures have also yielded promising results and continue to be used in the search for brain mechanisms underlying alcohol’s motivational effects. Moreover, although not discussed in this article, all of these procedures have been used or have the potential to be used to model alcohol craving and relapse to alcohol-seeking behavior after periods of abstinence. For example, self-administration models are being used to study the “alcohol deprivation effect,” a temporary increase in alcohol consumption observed following a period of forced abstinence (Heyser et al. 1999).

The distinction between appetitive and consummatory processes in alcohol self-administration (Samson and Hodge 1996) is a useful and important one for interpreting behavior in both models and especially for integrating findings across models. Although it is tempting to argue that one model or one particular procedure is better than another is, each model has distinct advantages and disadvantages on both methodological and theoretical grounds. In general, these models represent different approaches to understanding alcohol’s motivational effects and address different aspects of alcohol-seeking behavior and alcohol self-administration. In some cases, the nature of the model makes it difficult to address particular questions. For example, the need for a relatively long period of “initiation” in oral self-administration studies makes those procedures less well suited for studying initial sensitivity to alcohol’s motivational effects. At present, our knowledge of the exact role that alcohol’s motivational effects play in the development of excessive drinking and alcoholism in humans is too incomplete to eliminate any of these models from further consideration.

Finally, researchers must consider whether these animal models are relevant for understanding alcohol-induced motivational processes in humans. At one level, this seems possible. As already noted, the analogy between animals who orally self-administer alcohol and humans who voluntarily consume alcohol seems reasonable. The presumed role of alcohol’s motivational effects among humans is further strengthened by studies showing that among light-to-moderate drinkers, people who report positive motivational effects (e.g., increased elation, vigor, and arousal) are more likely to drink alcohol than those who report negative motivational effects (e.g., increased dysphoria and confusion, decreased elation) (de Wit et al. 1987). Of course, it will always be difficult to know how well an animal’s approach and avoidance of alcohol-paired cues correspond to verbal reports of positive and negative motivational states in humans. Ultimately, this issue will be decided by the utility of our animal models for identifying relevant genes and brain systems and for developing effective pharmacotherapies and behavioral interventions for alcoholism.
